# Editorial: Neurodegenerative Diseases: Looking Beyond the Boundaries of the Brain

**DOI:** 10.3389/fnins.2022.929786

**Published:** 2022-06-23

**Authors:** Gabriel Gutiérrez-Ospina, Claudia Perez-Cruz, Elena Zenaro, Marietta Zille

**Affiliations:** ^1^Laboratorio de Biología de Sistemas, Departamento de Biología Celular y Fisiología, Instituto de Investigaciones Biomédicas y Coordinación de Psicobiología y Neurociencias, Facultad de Psicología, Universidad Nacional Autónoma de México, Mexico City, Mexico; ^2^Center of Research and Advanced Studies (Cinvestav) of the National Polytechnic Institute, Mexico City, Mexico; ^3^Section of General Pathology, Department of Medicine, University of Verona, Verona, Italy; ^4^Division of Pharmacology and Toxicology, Department of Pharmaceutical Sciences, University of Vienna, Vienna, Austria

**Keywords:** neurodegeneration, peripheral alterations, body-brain interactions, outside brain boundaries, neurodegenerative diseases

Neurobiological wisdom has long entertained the notion that the brain has functional primacy over the body. Under this presumption, the body serves as the “machine” through which the brain manifests its “sublime workings” in the form of overt behavior. The body, nonetheless, does much more than only being the brain's marionette. Indeed, it “dialogues, cares, nourishes and may even condition” the brain so that its functional morphology stays operative throughout life until, eventually, with increasing age, the body-brain whole irreversibly decays as death approaches.

As the saying outlines, “a healthy mind is a healthy body” and numerous instances support this simple yet profound notion. The occurrence of reciprocal altruistic/instructive trophic interrelationships between the body and the brain has existed for decades (Purves, 1992). Scientific awareness about the overpowering influence the body's inner sensing has on our conscious cognitive abilities and behavioral manifestations has also increased greatly. Indeed, embodied cognition and emotions are no longer denied facts (Seth and Friston, 2016; Holzer, 2017; Nummenmaa et al., 2018).

Despite this information, many scientists still believe that the origin of neurodegenerative processes rests within the brain itself. However, this neurocentric view is confronted by relatively new evidence suggesting that neuronal deterioration may be ignited, and its progression fostered by processes ongoing outside the boundaries of the brain. These include, but are not limited to, an impaired cardiovascular system, infection, systemic inflammation, cellular senescence, altered trophic interactions, endocrine disruption, and gut dysbiosis (Preciados et al., 2016; Castillo et al., 2019; Limphaibool et al., 2019; Winek et al., 2022). “*Neurodegenerative Diseases: Looking Beyond the Boundaries of the Brain*” is a volume that looks at brain diseases as consequences rather than as the result of primary neurological causes.

Gut microbiota, for instance, modulate the immune system in a variety of neurological diseases (Cryan et al., 2020; Willyard, 2021). The imbalance of its composition, distribution, and metabolic activity may lead, as commented by Maiuolo et al., to anxiety, depression, autism spectrum disorder, and multiple sclerosis. Studying the crosstalk between neurons, mucosal immunity, and gut microbiota may help us improve therapeutic measures aimed at lessening these disorders. Dumitrescu et al. evaluated the presence of biomarkers of intestinal inflammation and barrier permeability in the peripheral circulation and stool samples obtained from Parkinson's disease subjects. The correlation observed supported a causal link between dysbiosis, enhanced inflammatory response, and the progression of neurodegeneration. Rydbom et al. demonstrated that Tau is capable of disrupting gut motility, microbiome composition, and innate immune response in Drosophila. This opens the possibility that peripheral tauopathy may alter the availability of the antimicrobial peptides that oversee the elimination of pathogenic microorganisms that might promote neurodegeneration. Lastly, sirtuins, a family of histone deacetylases, modulate genome stability, stress cellular response, and nutrient and hormone sensing in response to various metabolites that signal aging, obesity, and diabetes. Chandramowlishwaran et al. revised the novel role of sirtuins on enteric neuronal growth and survival, and propose sirtuins as novel modulators of the gut-brain axis.

In addition to dysbiosis, viral infections are presumed to cause neurodegeneration (Limphaibool et al., 2019; Shinjyo and Kita, 2021). With the novel SARS-CoV-2 infection, growing evidence supports infections as an etiological path to neurodegeneration and cerebrovascular disease (Wenzel et al., 2021; Douaud et al., 2022). Römer reviews the epidemiological and experimental evidence that links viruses and endogenous retroviruses to neuro-immune degeneration.

Lifestyle and metabolic alterations during midlife are considered important risk factors for developing Alzheimer's Disease (AD) (Livingston et al., 2017). Memory dysfunction in AD patients results from the brain and peripheral glucose resistance (Arnold et al., 2018). Wei et al. revise the multiple pathogenic mechanisms induced by insulin resistance that are implicated in AD and discuss the use of antidiabetic and anti-inflammatory drugs to delay the onset of neurodegeneration. Sim et al. offer a comprehensive overview of the potential role of dipeptidyl peptidase-4 inhibitor and sodium-glucose cotransporter 2 inhibitors, both used in type-2 diabetes patients, as repurposing drugs against AD based on their antidiabetic effects.

As already mentioned, several lifestyle factors during midlife are important modulators of dementia onset later in life. In particular, unhealthy dietary habits influence neurodegenerative diseases through cellular inflammation and increased oxidation (Tan and Norhaizan, 2019; Winiarska-Mieczan et al., 2020). Nassir et al. provide a persuasive and plausible explanation that supports the role of cellular-derived circulating microparticles released after consuming unhealthy diets in promoting thrombotic events throughout the microcirculation. When thinking about the etiology of neurodegeneration, these microparticles must then be considered as risk factors and biomarkers of the brain-heart-gut axis.

The erythroid 2-related factor 2 (NRF2) forms part of the molecular machinery that supports the body's antioxidant defense system. Petrillo et al. performed a family study of Friedreich's ataxia, the most frequent autosomal recessive ataxia in Western societies. Although all family members were affected by frataxin depletion, those having activation of NRF2 were asymptomatic. The authors propose that the constitutive upregulation of NRF2 keeps the antioxidant defense above the threshold, a circumstance that protects against progressive oxidative damage.

The glymphatic circulation modulates neuroinflammatory responses in neurodegenerative diseases. Natale et al. argue that, besides waste elimination, the glymphatic system may also function to connect the brain with the periphery. It then facilitates communication between the immune system and the brain, thus modulating the brain's immune surveillance and neuroinflammation. The functional disruption of the glymphatic system must then be considered when unrevealing the mechanisms of neurodegeneration.

It becomes more and more clear that neurodegeneration progresses silently for many years or even decades before it becomes diagnosed. Therefore, the identification of early clinical biomarkers is urgently needed. Zhang et al. suggest that evaluating retinal degeneration may be a gateway to understanding, monitoring the progression, and diagnosing Parkinson's disease, as visual symptoms appear early in the disease.

Taken together, this Research Topic highlights the relevance of studying the etiology of neurodegeneration beyond the boundaries of the brain. Notably, this compilation of articles describes how peripheral alterations can impact brain function and health. Further research is needed to better understand these paths to develop more effective and early treatment options to stop the growing number of patients suffering from neurodegenerative diseases.

## Author Contributions

All authors listed have made a substantial, direct, and intellectual contribution to the work and approved it for publication.

## Conflict of Interest

The authors declare that the research was conducted in the absence of any commercial or financial relationships that could be construed as a potential conflict of interest.

## Publisher's Note

All claims expressed in this article are solely those of the authors and do not necessarily represent those of their affiliated organizations, or those of the publisher, the editors and the reviewers. Any product that may be evaluated in this article, or claim that may be made by its manufacturer, is not guaranteed or endorsed by the publisher.
